# Retraction: FOXC1 silencing promotes A549 cell apoptosis through inhibiting the PI3K/AKT/hedgehog/Gli2 signaling pathway

**DOI:** 10.1039/d1ra90016a

**Published:** 2021-01-22

**Authors:** Laura Fisher

**Affiliations:** Royal Society of Chemistry Thomas Graham House, Science Park, Milton Road Cambridge CB4 0WF UK advances-rsc@rsc.org

## Abstract

Retraction of ‘FOXC1 silencing promotes A549 cell apoptosis through inhibiting the PI3K/AKT/hedgehog/Gli2 signaling pathway’ by Pei Wang *et al.*, *RSC Adv.*, 2018, **8**, 33786–33793, DOI: 10.1039/C8RA06041J.

The Royal Society of Chemistry hereby wholly retracts this *RSC Advances* article due to concerns with the reliability of the data. The images in the article, and the raw data provided by the authors, were screened by an image integrity expert.

In Fig. 1 and 2, there is no apparent background to the western blots. In the raw data provided by the authors, some background is displayed, but it is not clear whether this is genuine. For the FOXC1 panel in Fig. 1 and the β-actin panel in Fig. 2, the area immediately around the bands in the raw data has a different appearance to the rest of the raw data, indicating that the row of bands may have been placed on a false background.

Analysis of the western blots in Fig. 4 shows that all 4 panels have the same background, indicating that these images have been manipulated.

Given the significance of the concerns about the validity of both the data in the article and the raw data provided by the authors, the findings presented in this paper are not reliable.

The authors have been informed but have not responded to any correspondence regarding the retraction.

Signed: Laura Fisher, Executive Editor, *RSC Advances*

Date: 7^th^ January 2021

## Supplementary Material

